# Measurement of Intervertebral Cervical Motion by Means of Dynamic X-Ray Image Processing and Data Interpolation

**DOI:** 10.1155/2013/152920

**Published:** 2013-10-31

**Authors:** Paolo Bifulco, Mario Cesarelli, Maria Romano, Antonio Fratini, Mario Sansone

**Affiliations:** Department of Electrical Engineering and Information Technologies (DIETI), University of Naples “Federico II,” Via Claudio 21, 80125 Naples, Italy

## Abstract

Accurate measurement of intervertebral kinematics of the cervical spine can support the diagnosis of widespread diseases related to neck pain, such as chronic whiplash dysfunction, arthritis, and segmental degeneration. The natural inaccessibility of the spine, its complex anatomy, and the small range of motion only permit concise measurement in vivo. Low dose X-ray fluoroscopy allows time-continuous screening of cervical spine during patient's spontaneous motion. To obtain accurate motion measurements, each vertebra was tracked by means of image processing along a sequence of radiographic images. To obtain a time-continuous representation of motion and to reduce noise in the experimental data, smoothing spline interpolation was used. Estimation of intervertebral motion for cervical segments was obtained by processing patient's fluoroscopic sequence; intervertebral angle and displacement and the instantaneous centre of rotation were computed. The RMS value of fitting errors resulted in about 0.2 degree for rotation and 0.2 mm for displacements.

## 1. Introduction 

Neck pain is a common musculoskeletal problem experienced by the vast majority of the population [1, 2]. Alterations of cervical spine mechanics that compromise the stabilizing mechanisms of the cervical spine (such those originated by chronic whiplash dysfunction [3], arthritis [4, 5], and segmental degeneration [6]) can be possible causes of neck pain.

Detection of spinal instability (degenerative or traumatic) is based on accurate measurement of intervertebral kinematic [7]. In particular, forward displacement of the vertebrae greater than 3.5 mm and angle between adjacent endplates greater than 11 degree is regarded as a sign of instability [8] and indication for surgery.

Quantitative measurements of segmental kinematics also find use in the evaluation of cervical arthroplasty, assessment of disc prosthesis, postsurgery followup, and so forth [9–11]. 

In spite of their paramount importance in clinical application, accurate measurement of the intervertebral kinematic are hindered by the natural inaccessibility of the spine, the complexity of its anatomy, and physiology and the extremely small range of motion achieved at segmental level.

Although most of the injuries and degenerative pathologies of the cervical spine are associated with reduced mobility and pain, there is no gold standard for the measurement of the kinematics of the cervical spine, not even for the measurement of its range of motion as a whole. Many techniques were proposed to measure spine kinematics [12–15]. These techniques include those radiological (functional radiography, cine-radiography, stereo radiography, TC, MRI, etc.), those based on external markers motion tracking (electrogoniometers, inclinometers, electromagnetic markers, optical skin markers, etc.), those ultrasonic, and those invasive (e.g., insertion of rigid markers in the vertebra bones in the context of a surgical operation).

The simpler and less invasive methods (e.g., external goniometers, optical markers, etc.) can only provide appropriate information about the entire cervical range of motion, but they are unable to accurately assess intervertebral motion (relatively large errors are associated with the sliding between skin markers and bones). Despite patient's radiation exposure, the radiological methods are currently preferred for many diagnoses. In particular, functional radiography is the clinical standard to detect segmental instability and decide whether to perform a surgery. Intervertebral kinematics measurements are currently based on functional flexion-extension radiography [16–18]. However, this method involves the use of few, end-of-range spinal positions, while in-between intervertebral motion is disregarded.

It is worth mentioning that some authors suggested that disc degeneration may lead to abnormal location of intervertebral center of rotation while maintaining intervertebral translation and rotation within a normal range [16, 19, 20]. Other studies [21, 22] supported that the center of rotation is the most sensitive and specific measurement for detecting damage of intervertebral disc and facet joints. 

Because of the indirect methodology and the physician manual selection on radiographies, intervertebral kinematic measurements suffer from large inaccuracy. This is particularly true for the estimation of center of rotation because small errors in vertebra location result in much larger errors on the estimation of the center of rotation.

This study proposes a methodology for measuring the cervical intervertebral kinematics based on the processing of dynamic X-ray images able to provide objective measurements and a continuous description of spontaneous patient motion.

X-ray fluoroscopy can allow time-continuous screening of cervical vertebrae during spontaneous neck flexion extension. Fluoroscopy is based on high-gain image intensifiers to strongly reduce the X-ray radiation dose and allow prolonged recording, but it produces images with a much poorer SNR than conventional radiography. The position and orientation of each cervical vertebra were estimated frame by frame of the fluoroscopy sequence by means of an opportune time-varying image processing. The intervertebral kinematics was then estimated by combining the trajectories of two adjacent vertebrae. Clinically, relevant concise measurements were also computed as well as the trajectories of the instantaneous centre of rotation. Motion data were interpolated and filtered with nonfitting splines to obtain a time-continuous description of the joint kinematics. Approximation error analysis was also performed.

## 2. Materials and Methods

A 9-inche digital fluoroscopy device (Stenoscop, GE Medical Systems) was used for in vivo measurement. The X-ray tube parameters were adjusted for each subject; on average, they were set to 1 mAs and 50 kVp; the acquisition frame rate was set to 4 frame/sec; the focus-plane length was about 1 m. Digital radiological frames were acquired directly from the fluoroscopy device. Each image is memorized as raw image format (uncompressed), it is formed by 576 pixels, and luminance is encoded with 256 levels of gray, and the pixel size is 0.45 by 0.45 mm. The C-arm was set in horizontal position and the subject was put in, with his neck as close as possible to the image intensifier. Subjects were fastened to an adjustable-height chair by apposite belts in order to obtain shoulder stabilization. Subjects were instructed to spontaneously perform the maximum possible flexion-extension movement of their neck. Before recording, the subject became familiar with the assigned task in order to perform it correctly and enough slowly. The entire flexion-extension movement was performed in about 30 seconds. A calibration phantom was used to test for geometrical distortions and to measure image noise at different gray-levels.

Since vertebra registration is mainly based on matching of bones edges (a derivation operation is required on the images), noise reduction of the fluoroscopic images is of paramount importance.

In fluoroscopy, the numbers of X-ray photons are strongly reduced to keep patient's radiation dose acceptably low. The limited availability of photons per pixel generates the so-called quantum noise. Quantum noise is by far the most dominant noise in fluoroscopic images [23]. Quantum noise is a signal-dependent Poisson-distributed noise source [24], and its strength varies over the image depending on the local grey-level intensity. This noise cannot be considered space invariant, additive, Gaussian, and white.

An accurate noise model [25], considering Poisson's distribution, was held to quantitatively measure the fluoroscopic image noise. Preliminary, the relationship between noise variance and mean pixel intensity relative to the fluoroscopy device setup was estimated. Then, the fluoroscopy sequences were preprocessed by using an edge-preserving, adaptive average filter that incorporate the information of noise variance versus grey intensity [26]. By holding this information, noise suppression can be exclusively performed by averaging the only local data that have high probability to be included in the noise statistics. Filter operates both in space and time, preserving edges and motion [26].

**Figure 1:**
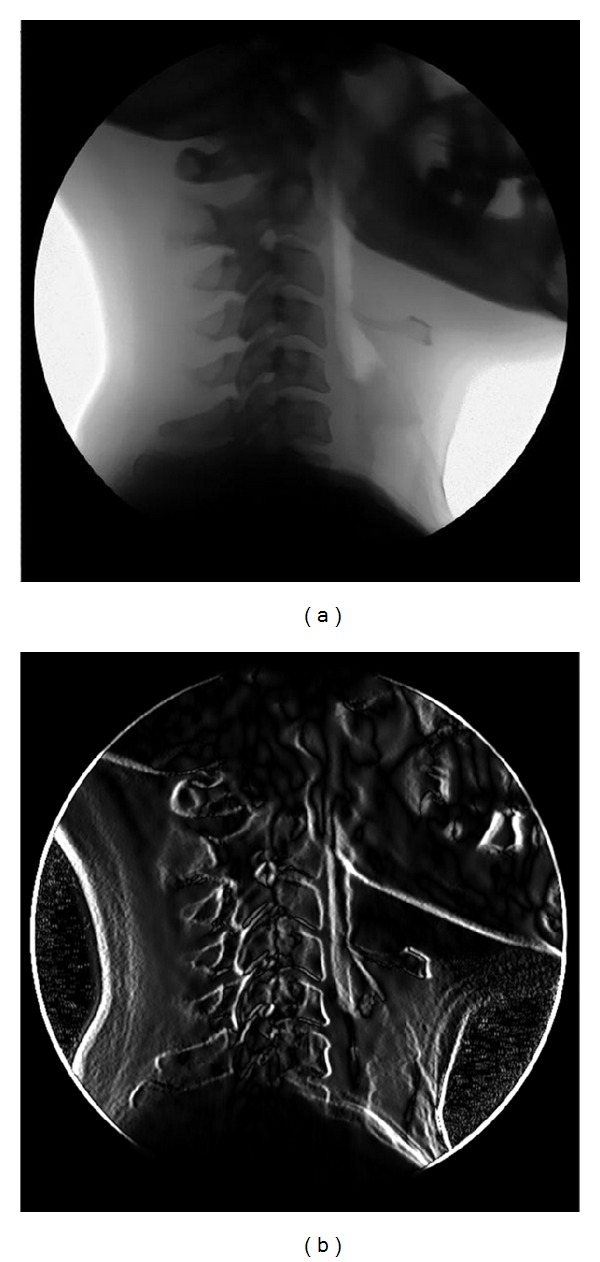
A prefiltered image of a fluoroscopic sequence (a) and the correspondent gradient image (b).

Vertebra tracking was achieved by image template matching [27, 28]. Cervical vertebrae were assumed to be rigid and the analysis was limited to the sagittal plane [29, 30] (see Figure 1).

A template of each cervical vertebra was chosen by selecting portion of the vertebra projection that does not superimpose with adjacent vertebra along with the whole the patient's motion. In particular, the cervical vertebra template included the anterior vertebral body cortex and the spinolaminar junction particularly visible on the radiographic projection of spinous process (the area of the facet joints was excluded). The inclusion of the posterior process in the template (in contrast with lumbar spine tracking [26], where only the vertebral body was considered) makes the error in vertebra positioning lower.

Vertebra tracking was achieved by matching the preselected vertebra template opportunely displaced and rotated on each of the image of the fluoroscopic sequence [31, 32].

The template matching was based on a particular image similarity index (GNCC), which combines the normalized cross-correlations of the horizontal and vertical gradients of the fluoroscopic image. The GNCC index was obtained according to the following formula:

(1) GNCC(i,j)=12·∑(x,y)∈Tgx(i+x,j+y)·tx(x,y)∑(x,y)∈Tgx2(i+x,j+y)·∑(x,y)∈Ttx2(x,y) +12·∑(x,y)∈Tgy(i+x,j+y)·ty(x,y)∑(x,y)∈Tgy2(i+x,j+y)·∑(x,y)∈Tty2(x,y),

where *g*_*x*_ and *g*_*y*_ are the components of the gradient vector in the horizontal and vertical directions of a generic fluoroscopic image and *t*_*x*_ and *t*_*y*_ are the components of the gradient vector relative to the template; the summations are extended to the only pixels, of coordinates (*x*, *y*), belonging to the template. It is worth noting that this expression for the cross-correlation index not only takes into account the product of the gradient magnitudes but also performs a scalar product between the gradient vectors. This improves accuracy of vertebra location with respect to the simple normalized cross-correlation image matching.

Since each vertebra can be spatially translated and rotated in between two fluoroscopic images, the maximum of the GNCC index was searched in the three parameter spaces: *x*-displacement, *y*-displacement, and rotation angle. This was obtained by progressively rotating the template with 0.1 degree increments and repeatedly computing the GNCC index. The coordinates of the global maximum of the GNCC index estimate the template displacement, while the angle corresponding to that maximum is held as the template rotation. Furthermore, 2D cubic interpolation of the GNCC function provided a subpixel accuracy for the vertebra displacement.

**Figure 2:**
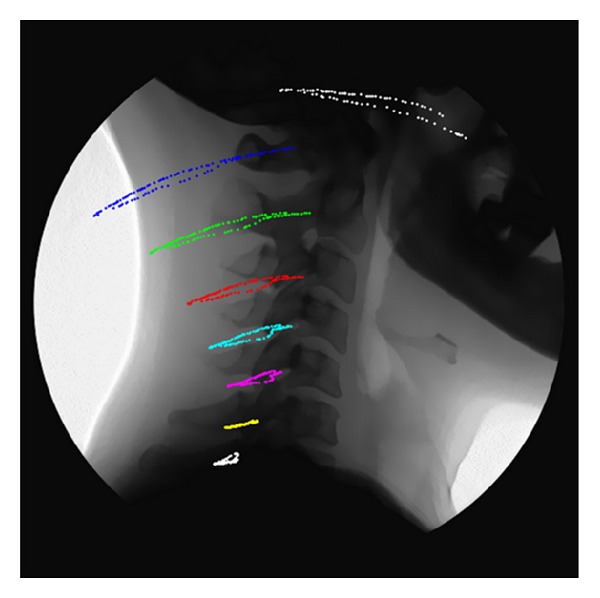
Absolute trajectories of vertebrae during flexion-extension.

At the end of the vertebra tracking procedure, the estimated *x*- and *y*-displacements and angles of rotation of a selected vertebra are available for all the frames of the fluoroscopic sequence. These three parameters, over time, completely describe the planar, rigid motion (i.e., the trajectory) of each cervical vertebra (see Figure 2).

From these data, the intervertebral description of motion was obtained, that is, the trajectory of the upper vertebra with respect to the lower vertebra considered motionless.

In particular, the intervertebral angle of rotation *α*_*IV*_ was given by

(2) $$\begin{array}{c} \alpha_{IV}=\alpha_{UV}-\alpha_{LV} \end{array}$$

and the intervertebral displacements (*x*_*IV*_, *y*_*IV*_) were given by

(3) $$\begin{array}{c} \left(\begin{array}{c} x_{IV}\\ y_{IV} \end{array}\right)=\left(\begin{array}{cc} \cos\left(-\alpha_{LV}\right) & -\sin\left(-\alpha_{LV}\right)\\ \sin\left(-\alpha_{LV}\right) & \cos\left(-\alpha_{LV}\right) \end{array}\right)\cdot \left(\begin{array}{c} x_{UV}-x_{LV}\\ y_{UV}-y_{LV} \end{array}\right), \end{array}$$

where *α*_*UV*_ is the angle of rotation of the upper vertebra, *α*_*LV*_ is the rotation of the lower vertebra, (*x*_*UV*_, *y*_*UV*_) are the *x*- and *y*-displacements of the upper vertebra, and (*x*_*LV*_, *y*_*LV*_) are the *x*- and *y*-displacements of the lower vertebra.

The intervertebral discrete-time data were interpolated by quintic nonfitting spline (similarly to [33]) providing both a continuous-time description of motion and a low-pass filtering of the experimental data. The intervertebral discrete-time kinematic signals can be considered as a summation of the true kinematic signal (i.e., intervertebral motion) and noise (i.e., measurement error). Since true intervertebral motion can be only gradual and smooth, it is band-limited. On the contrary, measurement errors depend on several factors (e.g., imperfections in the algorithms, computation approximation, and quantization errors) and they can be considered as additive, Gaussian, and white (i.e., band-unlimited). Therefore, the lower frequency part of the signals is associated with the motion signal, while the remaining (high-frequency content) is exclusively related to noise.

Fitting errors (i.e., residuals) of the spline interpolation were analyzed as they represent a quantitative index of the precision of the measurement made. The change-sign and Box-Pierce tests for whiteness were performed to ensure that the measurement errors were uncorrelated (i.e., representative of random noise and not motion).

Instantaneous centre of rotations (ICRs) were also computed but only for absolute angular velocities greater than 1 degree per second.

## 3. Results

**Figure 3:**
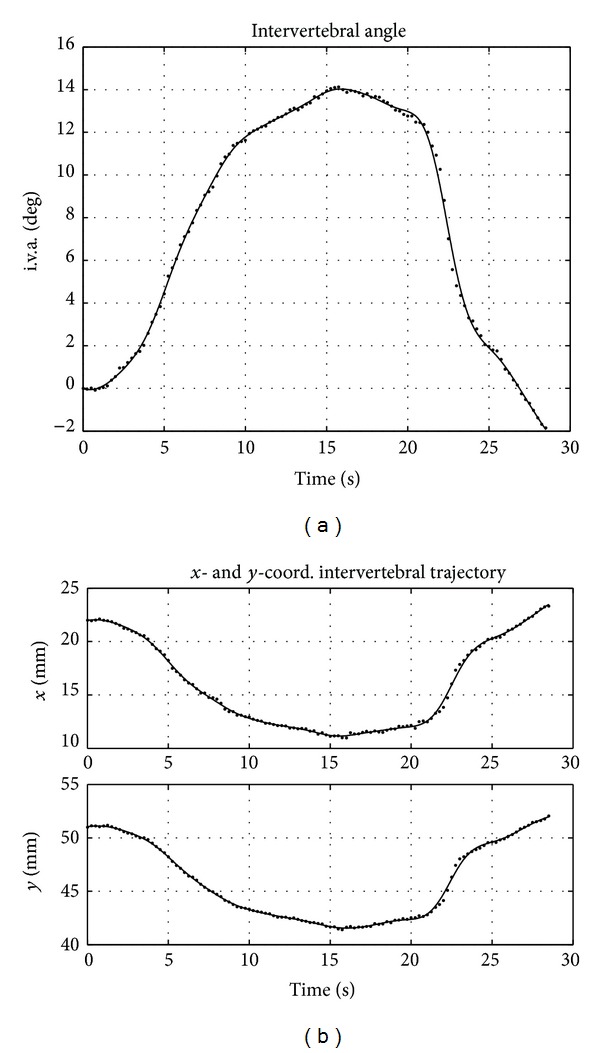
Segment C5-C6. (a) Intervertebral angle measurements (dots) and spline interpolation (cont. line). (b) Intervertebral displacements.

Once the absolute cinematic is computed (see Figure 2), the intervertebral measurements were computed. As an example, the intervertebral kinematics of the segment C5-C6 is presented in Figure 3.

Discrete measurements are depicted as dots, while the spline polynomial interpolations are shown as continuous lines. 

**Figure 4:**
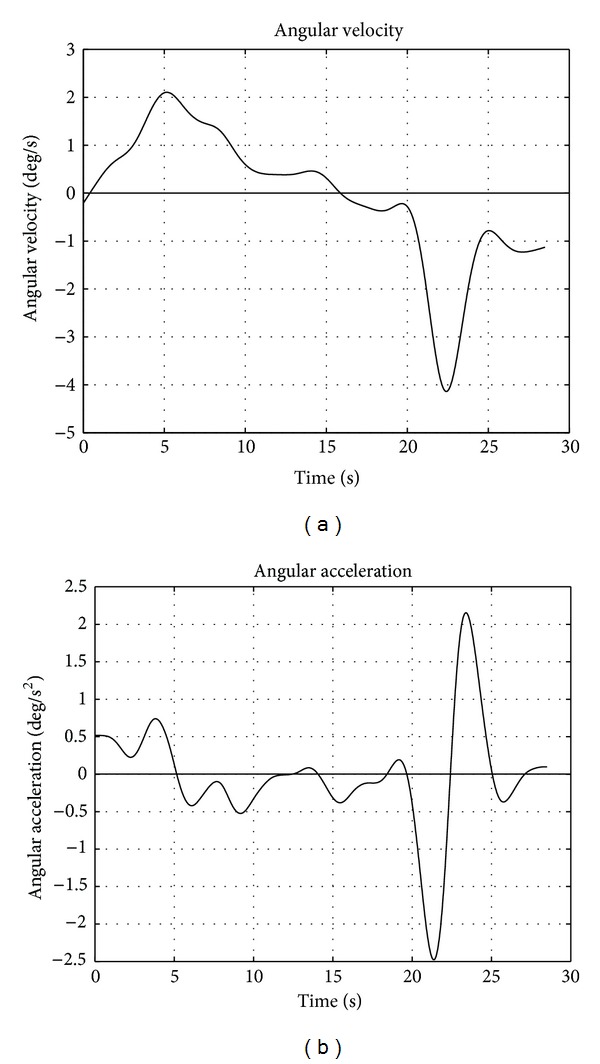
Intervertebral angular velocity (a) and acceleration (b) computed by deriving the polynomial spline approximation of the rotation.

The extension phase develops in the time interval 3–9 s, while the flexion mainly at 21–24 s. Angular and linear velocities and acceleration were analytically calculated from the coefficients of the interpolating polynomial (Figure 4). Angle and displacement measurements can be considered as a superposition of the true kinematic signal (i.e., intervertebral motion) and noise (i.e., measurement error). The true kinematic signal can be considered band-limited (motion can only be gradual and smooth). Conversely, measurement error depends on several factors and can be generally considered as additive and white (i.e., uncorrelated, band-unlimited). Therefore, the lower frequency part of the signals (spline interpolated) is mainly associated with motion, while the remaining is associated with noise. 

**Figure 5:**
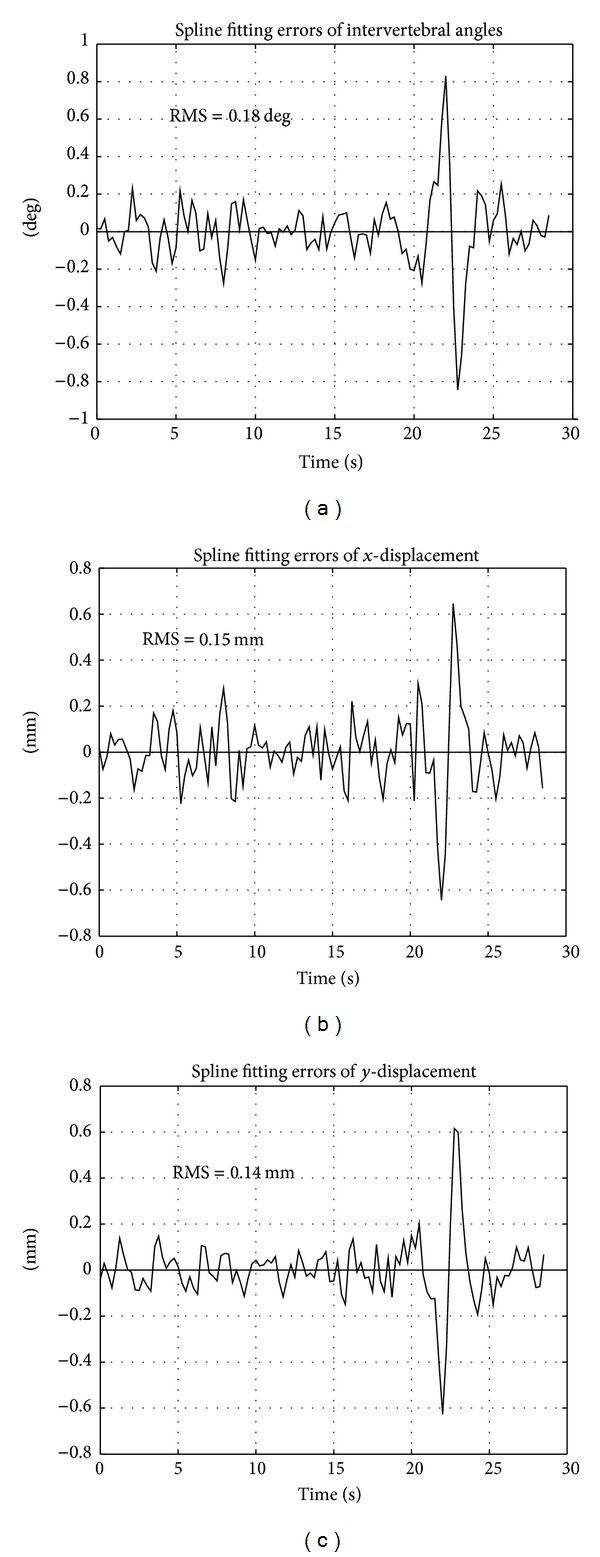
Fitting errors of spline interpolation: (a) intervertebral angle; (b) intervertebral *x*-displacement, and (c) *y*-displacement.

Figure 5 represents the spline fitting errors (i.e., residual of the interpolation) for the intervertebral angle and the displacements. The whiteness test of the measurement errors was verified (with a significance level of 0.05) for spline smoothing parameters, *P* > 0.95. However, a larger error in correspondence of the flexion is visible, where the angular velocity resulted about in double of that corresponding to extension.

RMS values of the residuals resulted in 0.18 degree for the intervertebral angle and 0.15 mm and 0.14 mm for the intervertebral *x*- and *y*-displacements, respectively.

**Figure 6:**
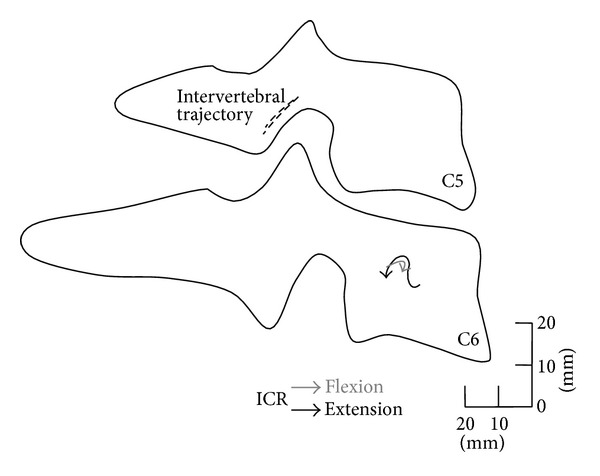
ICR trajectories during flexion and extension.

As an example, trajectories of the instantaneous centers of rotation of the segment C5-C6 are represented in Figure 6 superimposed on a schematic profile of the vertebrae.

During flexion (time interval 21–24 s), the ICRs move somewhat anteriorly, while in extension (3–9 s), move posteriorly. ICR trajectories result placed in the same location, they found in previous studies [20] for the finite centers of rotation (i.e., computed between two extremes of motion).

## 4. Discussion

Intervertebral kinematics closely relates to the condition of the soft tissue (disk, ligaments, etc.) intended to constrain segmental motion to maintain stability. Despite its importance, intervertebral kinematics is difficult to measure in vivo: direct measurements are not clinically viable, and little errors in estimation of vertebrae positioning may cause large relative errors in intervertebral measures. By means of fluoroscopy, it is possible to describe the whole progress of intervertebral motion in the plane of view. Template matching techniques can provide estimation of vertebra position in each frame. Then, spline interpolation provides both noise reduction and continuous representation of motion. Analysis of the measurement error shows their uncorrelation. 

It is worth to underline that the sampling frequency (i.e., the frame rate) has to respect Nyquist's theorem. Therefore, the movements of the patients should be enough slowly and smooth. At the moment, this technique seems inadequate to measure intervertebral kinematics (e.g., disk deformations) during vibrations [34, 35] and rapid mechanical stress or shock (as in case of car accidents, which are common cause of cervical whiplash). In these cases, the hypothesis that the high frequency components of the measured kinematics are exclusively related to noise is not fulfilled.

Previous studies [22] pointed out that the intervertebral centre of rotation is much more sensible to mild degeneration of disk and ligament. Most of the literature presents the finite centre of rotation (easier to compute) that represents only an approximation of the ICR. ICR trajectories can provide better understanding of the segmental motion in vivo.

Accurate measurement of intervertebral kinematics can offer an objective diagnostic tool to evaluate mechanical alteration of cervical segments and also can support evaluation and settings of different prostheses even during the implantation.
